# Welfare Quality of Breeding Horses Under Different Housing Conditions

**DOI:** 10.3390/ani9030081

**Published:** 2019-03-05

**Authors:** Silvana Popescu, Eva A. Lazar, Cristin Borda, Mihaela Niculae, Carmen D. Sandru, Marina Spinu

**Affiliations:** Faculty of Veterinary Medicine, University of Agricultural Sciences and Veterinary Medicine, 400372 Cluj-Napoca, Romania; lazarevaandrea@gmail.com (E.A.L.); cborda@usamvcluj.ro (C.B.); niculaemihaela1@gmail.com (M.N.); sandranac@gmail.com (C.D.S.); marina.spinu@gmail.com (M.S.)

**Keywords:** Brood mares, breeding stallions, horse health, horse behavior, welfare score

## Abstract

**Simple Summary:**

Depending on their use, horses are exposed to specific welfare risks. The aim of this study was to gain an insight into the welfare of breeding mares and stallions, in different types of housing, a topic which little has been written on. The assessed breeding horses were included in one of four welfare categories on the basis of a numerical welfare score calculated by the assessment of 30 management and animal-related indicators (health and behavior). The study also evidences the deficiencies and negative effects on horses’ welfare from the tie-stalls system, which is still used in some countries. This housing type is linked to increased risks of respiratory and locomotive problems, which have a significantly higher prevalence in the tie-stalled stallions than in mostly freely kept mares. The overall welfare categories recorded showed better welfare in the mares (“enhanced” and “excellent”) than in the stallions (“acceptable” and “enhanced”). Accordingly, it can be concluded that positive changes in housing management, such as free housing with the use of boxes, could improve the welfare quality of breeding stallions.

**Abstract:**

This paper investigates the effect of different housing conditions on the welfare quality of breeding horses. Using a welfare protocol that included health and behavioral parameters, 330 stallions (kept in tie-stall housing) and 365 broodmares (kept in extensive, mostly free housing) were assessed. The horses were categorized into four welfare categories (“not classified”, “acceptable”, “enhanced” and “excellent”), according to an individual welfare score calculated for each horse. The prevalence of stallions with dyspnea, tendon and joint swellings, abnormal gait and abnormal hoof horn quality was significantly (*p* < 0.05) higher than that of the broodmares. No significant difference (*p* > 0.05) was found in the human-related behavioral response of the two categories of breeding horses. The median individual welfare scores were significantly higher (*p* < 0.05) in the broodmares than in the breeding stallions. The mares had “enhanced” and “excellent” welfare, while the stallions had “acceptable” and “enhanced” welfare. The results revealed differences in the horses’ welfare quality for the different housing conditions. Accordingly, it can be concluded that positive changes in housing management, such as free housing with the use of boxes, could improve the welfare quality of breeding stallions.

## 1. Introduction

Housing conditions for horses have changed throughout history. Today, we are still trying to find improvements and identify systems that would better balance the benefits for the horse, economic incentives and recommendations based on current knowledge. In some parts of the world, stall-tying is considered as “a common method, historically used for stabling cavalry horses” [1]. In Eastern Europe, especially on large breeding farms, this practice is still used, mostly for breeding stallions. In addition, the mares, fillies and colts are kept stall-tied during the night and also during part of the cold season. In contrast, most of the horses in Western countries are individually housed in boxes that can allow a range of types of social contact. Even where the mares are turned out alone in a flat, non-stimulating environment [2], at least they have access to free movement. 

Around the world, stallions are housed in single stalls, which are usually more restrictive than the housing of mares, geldings or foals. Box housing provides better comfort for resting and addresses the owners’ fear that the stallions will fight and injure each other [2] but it limits to a great extent their opportunities for social interaction and locomotion [3].

The practice of keeping stallions in solitary confinement causes significant stress and increases aggressive behavior [4,5]. Free exercise is important for the horses’ overall health and fitness [6,7,8]. In addition to the physical benefits, exercise, and especially free exercise around other horses, is very important for the development and welfare of the animals’ mental status [9]. Restriction of social interaction and locomotive behavior can reduce animal welfare [10] in this gregarious species. Keeping horses stabled and using them for reproduction purposes requires consideration on how the environment and management practices will affect their physical and mental welfare. The housing system offers complex environmental conditions for the everyday living of the housed animals. Therefore, one of the main factors in the welfare and performance of the horses is the optimal facilitation of their behavioral needs. To increase the safety and performance level of horses, their housing conditions should closely mimic the natural conditions of these animals [2]. 

There is a general lack of data about the welfare of breeding horses. Campbell [11] suggests that this could be rectified by the addition of such horses as a separate category to ongoing data collected about general horse welfare. This approach is justified by the fact that different horse categories are subject to specific risks for poor welfare, depending on their use. 

The aim of this study was to gain an insight on the effect of different housing conditions on the welfare quality of breeding horses. 

## 2. Materials and Methods 

### 2.1. Animals

The breeds of the horses (330 stallions and 365 broodmares) included in this study were Furioso North-Star (22.16%), Lipizzan (45.9%) and Romanian Draft Horse (31.94%), and they were kept in four different breeding farms in Romania (farm A: 76 stallions and 62 broodmares, farm B: 84 stallions and 120 broodmares, farm C: 96 stallions and 86 broodmares and farm D: 74 stallions and 97 broodmares). All the horses in this sample were purebred registered equine. 

The stallions were housed tethered throughout the year. Being individually stall-tied, they could not have physical contact with each other, but all had all the other possibilities of contact (auditory, visual and olfactory). The stallions were fed by hand, with four hay bales and two daily concentrate meals. Watering was done through automatic waterers for most horses and manually, four times a day, for the others. The entire daily human handling of the adult stallions, except necessary contact with humans during feeding, watering and bed cleaning, consisted of grooming and inconsistent hoof cleaning. None of the stallions were regularly ridden or used to drive carts. Even if each farm had several (ten or less) outside paddocks for individual turnouts of the stallions, none of the studied farms had a regular schedule for providing access to free exercise for the animals. The mares were generally free on pastures, tethered when fed and during the night in winter. Similar to stallions, watering was done four times a day for about a quarter of them and the others had access to automatic drinkers in the barns and water troughs in the paddocks. 

The housing and horse management conditions described above were permanent, as they have not changed significantly in the past 50 years.

All the procedures involving the animals were carried out in accordance with the ethical guidelines of the Romanian National Animal Protection Law [12].

### 2.2. Welfare Assessment

The welfare assessment of the horses was performed during the warm season, using a protocol based on the five freedoms previously described by Popescu and Diugan [13,14]. Scores were assigned to each parameter by the scoring system published by Popescu and Diugan [14]. Table 1 shows the welfare parameters assessed and their scoring.

All the stallions and mares in the visited farms were assessed once (a total number of 695 horses) by pairs of experienced researchers (one was assessing, the other recording the scores, then the roles were reversed), with an inter-assessor reliability of at least 80%. The assessments were undertaken during several days in the afternoons (between 2 p.m. and 5 p.m.), outside of the horses’ feeding time. All the studied horses were fire branded. The brands were recorded for identification, to avoid repeated assessment of the same individual and to ensure that all the horses in a group were assessed. This was especially helpful for the mares assessed in the paddocks or pastures. 

The freedom from fear and distress was assessed first, as the researcher approached the horse, to evaluate how the horse reacts in the presence of the unknown assessor. The next parameter to be assessed both visually and by palpation was the body condition score (BCS), and then the other parameters, which are meant to describe the freedom from discomfort and the freedom from pain, injury or disease. Each horse’s gait was evaluated at the end of the assessment. 

To establish the scores for some parameters, the caregivers or responsible veterinarians were interviewed, for example to score water provision where no automatic drinkers were installed, dental check and to determine access to exercise for the stallions in tethered housing. 

To assess the quality of the horses’ welfare, a score was calculated for each animal. This individual welfare score was computed by adding up the scores obtained for each parameter. The maximum possible score was 50. The higher the score obtained, the more appropriate the welfare of the horse. 

### 2.3. Overall Assessment

After calculating the individual welfare scores of each horse, four qualitative welfare classes were established to determine the qualitative significance of the numerical results (Table 2).

The ‘excellent’ category was for the horses recorded as having maximum scores for all or almost all parameters, meaning that they had the best resources and they benefit in the best way from their living environment. The ‘enhanced’ category comprised individuals that had satisfactory welfare status and were kept in overall good conditions with only few areas that needed some improvements. Horses in the ‘acceptable’ category were those doing fairly well in their environment, but having limited resources, or their situation required intervention in the near future to improve their lives. The ‘not classified’ category was meant to describe horses with poor welfare, who were kept in unacceptable conditions, who were constantly struggling to achieve well-being in their environment. For these individuals, immediate intervention was necessary to reduce their suffering, improve their welfare and to offer proper remedies with the maximum possible attention.

### 2.4. Statistical Analyses 

Statistical analyses were carried out using the SPSS (version 17, 2010, www.spss.com) software program. The calculated descriptive statistical parameters included the mean, standard error of the mean, median, minimum and maximum. The results obtained for the welfare parameters were expressed as percentages. All the data were tested by using the Kolmogorov–Smirnov test for normality distribution. The Mann–Whitney test was used to compare the non-parametric data relating to different breeding categories. The level of statistical significance was set at *p* < 0.05.

## 3. Results

### 3.1. Welfare Assessment and the Welfare Score 

Table 3 records the prevalence of the parameters assessed for the investigation of freedom from hunger and thirst of the stallions and broodmares. No statistically significant differences were found regarding these parameters between the breeding horse categories. 

None of the horses had hip point lesions and scars or thickened skin with missing hair. Only a small percent of stallions (8.18%) had faecal soiling on the rump and ventral-lateral abdomen, the difference between the two categories of horses being insignificant (*p* > 0.05).

The prevalence of parameters assessed for the investigation of freedom from pain, injury or disease in each breeding horse category is shown in Table 4. A higher frequency of health problems was noted in stallions. For some health parameters (swollen tendons/joints, hoof horn quality, gait, dyspnea) significant (*p* < 0.05) differences were recorded between the two breeding horse categories. 

The prevalence of the behavioral parameters investigated in each breeding horse category can be seen in Table 5 and Table 6. 

The general attitude of the stallions and broodmares was similar, with the majority of the animals being alert (Table 6). 

In the behavioral reactions of the horses in the three tests performed, fear was more often noticed in the mares and indifference in the stallions. In all tests, the highest frequency of the friendly response to human presence was recorded in the stallions. 

The descriptive statistical parameters of the individual welfare scores in the stallions and broodmares are shown in Table 7. The medians of the welfare scores of the broodmares were significantly higher (*p* < 0.05) than those of the stallions. 

### 3.2. Overall Assessment

**Figure 1:**
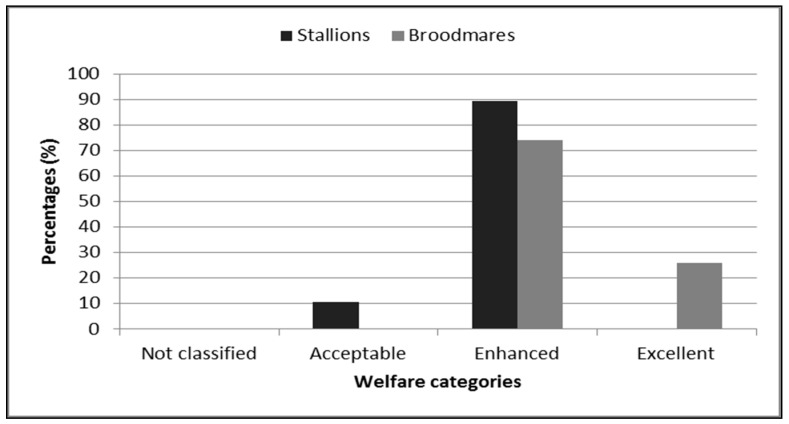
Classification of the stallions and broodmares in different welfare categories, based on the final score.

The welfare quality of the stallions and broodmares is presented in Figure 1. Based on the scores obtained, the stallions were classified in the acceptable (10.6%) and enhanced (89.4%) welfare categories, and the mares were classified in the enhanced (74.2%) and excellent categories (25.8%), respectively. 

## 4. Discussion

The frequency of mares with a low body condition score (BCS) was higher than that of the stallions, a result in line with that which was reported by Sanmartín-Sanchez et al. [15] in Spain. The causes for weight loss in horses can be a lower energetic value of the feed than their needs (both quantitatively and qualitatively), dental problems, pain, internal parasites, neoplasms and a variety of chronic diseases [16,17]. A low BCS can have detrimental effects on animal welfare, being associated with disease symptoms and behavioral disorders [13,18].

In this study, some stallions did not have unlimited access to water, which is contrary to existing welfare recommendations. Popescu and Diugan [14] signaled this problem in other equine facilities in Romania as well. The importance of optimal water consumption cannot be overlooked. Some of the authors [19] considered that freedom from thirst was more important than freedom from hunger. Thus, consideration should be given to all factors and causes limiting adequate water intake and hydration in horses. Besides the need to provide constant access, the quality of the water provided has to be in line with potability standards. Even where access is provided, pollutants can change the taste of the water and decrease water consumption. According to Cymbaluk [20], the water provided for horses should be chemically and microbiologically tested to be safe, in ad libitum or sufficient ad libitum amounts to satisfy each individual’s needs. This is especially important for the permanently stabled stallions in this study who are fed only with hay, irrespective of the season [20], as they need more water to stay properly hydrated.

The direct parameters assessed for the freedom from discomfort show a generally good situation for the studied stallions and mares. The assessment of barn hygiene based on the body hygiene of the animals is a well-established evaluation method which has been long used for dairy cows [21,22,23]. Despite the widespread use of these parameters in cows, the reference literature did not suggests its use for horses. Unlike dairy cows, which are often housed in intensive production systems, where the numbers of the cows per working personnel is high, this is not the case in equestrian facilities, where horses are fewer in number compared to cows. Thus, they can be groomed and look clean, even if their environment is not, lowering the accuracy of this indicator. When not housed, horses keep clean, because they always choose clean and dry resting surfaces or even abandon laying down if the surface available is wet and dirty [24]. With this in mind, we considered that the presence of manure on the assessed horses’ bodies would strongly indicate poor environmental hygiene in their living area. The results obtained in this study confirmed this, as only the stallions permanently housed had their bodies dirty with manure. 

Health problems were more frequent for stallions than for mares, probably because of their different housing conditions. Discomfort or painful conditions can arise through injury associated with inadequate housing, management and equipment [25].

A recent study which investigated the interrelations between the welfare parameters in working horses sustains that a healthy hair coat is a valuable indicator of good welfare [26]. Even if the general aspect of their hair is closely related to the systemic health of the animal, its partial destruction can be the consequence of management factors or displayed behaviors. 

Lesions were more frequent in the stallions than in the broodmares (Table 4). Where direct physical contact is possible, especially when mares are around, adult stallions may attack each other, resulting in injuries to each other. Intra-species aggressiveness is much lower in the group of broodmares [27], as the individuals in the horse groups with established hierarchy show the lowest degree of aggressiveness that is needed in a given situation [28]. Increased lesions in stallions could also be linked to tethering practices [15].

The swelling of tendons and/or joints was found more frequently in stallions, compared with the broodmares. It is well known that swelling occurs as part of an inflammation. Leg inflammations in horses are most often of a traumatic origin or due to mechanical overload. For tethered stallions, injuries may occur if they hit the partitions of the barn or the rails meant to separate them from each other. Mechanical overload in their situation is not due to fast work or stepping on uneven surfaces, but is mostly caused by standing without stimulation of the blood and lymph circulation, as they have almost no access to free movement. Unbalanced hooves caused by uneven flooring and uneven growth add improper weight bearing and contribute to the occurrence of edema on their legs. 

The health of the hooves was assessed by a variety of parameters (Table 4). A high proportion of lameness in working horses originates in the hoof, partly caused by incorrect trimming and shoeing [13,29]. Daily hoof-care and the technique and frequency of hoof trimming has a great impact on all the hoof health parameters assessed in the study [29]. Improper length of the hooves (too long or too short) was the most frequent problem within this group of parameters and abnormal hoof-horn quality was significantly higher in the stallions than in the mares. Most hoof-related parameters in the stallions could be affected by long-term standing on wet and soiled bedding, and also by poor peripheral circulation, which was not supported by weight bearing during exercise. The significantly higher percentage of stallions with abnormal gait was in line with the results of other studies [14,15], and it can be explained by the tethered management that they could be subjected to and a long term lack of exercise, leading to muscular atrophy or the underdevelopment of certain muscular groups, especially in the young horses. Skin lesions, lameness and hoof abnormalities are problems widely reported in working equines [14,30,31,32].

The parameters for the health of the respiratory system were assessed having regard to diseases such as recurrent airway obstruction. Genetic and environmental factors interact in the development of this disorder [33], with the exposure of sensitive horses to airborne allergens having a triggering role. Clinical signs in horses with this disease include difficulties in breathing, chronic coughing and purulent nasal discharge [34,35]. The season plays a considerable role in respiratory disorders, with increased risk factors in winter and spring compared to summer [36]. Inflammatory airway disease was more frequently seen in horses kept outside in winter than in stabled horses [37]. Within the breeding horse categories assessed, only the stallions presented respiratory problems (Table 4). The explanation for this result would be the fact that they were spending the longest time in barns, tethered, being the most exposed to airborne allergens and pollutants. However, the building style of the barns (high roofs and exhausting chimneys), as well as frequent evacuation of the manure, represented various ways to avoid the accumulation of gaseous air pollutants and inhalable solid particles in the air at the breathing level of the housed horses. It is reported that inflammatory airway conditions are rare where animals are outside all year around, but are common when horses are stabled indoors [34]. The other health related parameters showed no unusual aspects in any of the horse categories assessed. 

As for the freedom to express normal behaviors, the stallions were disadvantaged because of how they were managed. With the stallions, tactile contact was limited by tethering to avoid fighting. Social isolation can lead to the development of behavioral problems in horses: They need daily contact with others of their own kind for a normal life and mental wellbeing [9,38]. Horses housed with conspecifics are less prone to develop abnormal behaviors [5,39], and increasing the possibility of visual and generally close contact with their neighbors can be of benefit [40]. The need for free exercise in horses is demonstrated by the compensatory increase in their activity level when the animals are released after even a short period (a few days) of free movement restriction [41]. Horses confined for prolonged periods may become increasingly frustrated by a lack of exercise, and they are likely to exhibit other adverse effects on their social behavior as well. No stereotypies were seen in the breeding horses assessed, probably because even in the absence of physical contact, the stallions could still see, hear and smell each other. It has been suggested that close contact of the horses with their neighbours, through grids for example, is associated with lower risks of stereotypical behaviors [40,42]. These findings support our results. 

The human-horse relationship was assessed using observation and three practical tests. An apathetic general attitude and a lack of response to stimuli from the environment often occurs in sick horses and in those animals which are overworked to the level of becoming “switched off” [43]. According to Hausberger et al. [44], the lack of responses of horses may indicate a possible discomfort that, if not removed, can affect their welfare. The proportion of apathetic horses was lower than that recorded by Sanmartín-Sanchez et al. [15] in Spain. The fact that this study recorded depressed animals only in the breeding stallion category could be related to their tethered housing without access to free exercise. The breeding stallions might have learned that, irrespective of their behavioral choices, they do not have any escape from their situation. Thus, the distress experienced by these animals could be considerably high, having an impact on their individual welfare. 

The human-horse relationship was generally not appropriate either, as almost half of these animals showed fear towards the assessors. From a welfare point of view, a good human-horse relationship is a key factor in the safe use of the animal [45]. Even if no significant differences were found in the behavioral reactions of the breeding horses between the two investigated categories, the results indicated a higher frequency of the indifferent and friendly responses in the stallions than in the mares. In all the behavioral tests, the mares were seen to be more fearful than the stallions. The almost continuous human presence and very frequent contact with human personnel is unavoidable when the horses are kept tied, and this could explain these results. Sankey et al. [46] have shown a direct correlation between repeated positive interactions between horses and humans and the positive attitudes of horses towards humans. The horses are able to draw general conclusions about horse-human interaction from their positive experiences with one person. The worse the connotations of interactions with people for the horse (stressful, scaring or painful), the more the horse will avoid similar interactions and display more intense fear reactions [45]. The assessed horses showed increasingly more fear as the assessor was getting closer to and finally touched them during the behavioral tests. This was possibly influenced by the attitude of the people with whom the horses had previous experiences. Recognizing this negative mental state is important, because fear and stress (both chronic and acute) can impact the health and welfare of the animals [47]. Moreover, the horses’ fear most commonly is correlated with aggressiveness towards people [47]. This is in accordance with our study: The mares displayed both fear and aggressiveness in the touching test. Besides this, the higher percentage of aggressive responses in the mares than in the stallions (Table 6) is in disagreement with the results of other researchers, who sustain that horses housed in paddocks are less aggressive towards humans and develop less behavioral disorders than those housed in boxes [48,49]. However, the causes of stress in large groups of breeding horses is multifactorial, as evidenced by the fact that even the broodmares maintained on pasture show a high incidence of gastric ulcers [50].

The results regarding the welfare score are in line with those reported recently by Popescu and Diugan [14]. The higher mean welfare score of broodmares can be explained by their different housing conditions, with access to pastures, free exercise and social contact. Some studies [51,52] have provided welfare scores for working horses. Our results cannot be compared with these because the parameters and calculation method are different. 

There are no available results regarding similar categorizations in welfare categories of breeding horses. The overall situation of the stallions showed more deficits compared with the broodmares. Lower scores (26–35 out of 50) were obtained for the stallions than for the mares and these indicated only acceptable welfare. The higher scores (between 46 and 50 out of 50) were recorded only in the broodmares. The better welfare of the mares in this study is explained by the mares’ ability to successfully use the resources they were provided with. Because the individual welfare scores are a summation of all the indicators that assess all the five freedoms, the higher scores indicate a better general situation regarding the welfare of the horses. 

To increase the prevalence of high scores, better management practices are needed for the keeping and caring of the horses, and also a better provision of resources to fulfill their species-specific needs.

A limitation of the present study is that by summing up the scores, the low values obtained for a parameter are compensated by the high values of other parameters, resulting in a misleading equivalence of scores. A way to avoid this inconvenience would be to calculate the weighed scores, as the Welfare Quality Assessment protocols [19] recommend for other species. The simple calculation method was chosen intentionally for this study, to facilitate ranking the animals according to their overall welfare status. The assessment of the animals and the calculation of the scores were performed in a standardized way, and it was considered that these individual scores could be used for statistical processing and to draw objective conclusions. 

## 5. Conclusions

The results revealed differences in the horses’ welfare quality for the different housing conditions. Positive changes in the housing management, such as free housing with the use of boxes, could improve the welfare quality of breeding stallions.

The major welfare problems identified in the stallions (i.e., respiratory and locomotive disorders) seem to be related to tie-stall housing and especially management that allows very little access to free movement. The assessment could have shown different results, even for the horses kept in this housing system (i.e., tie-stalls), with a better management of the stallion’s access to free exercise. The other main welfare problem identified was the lack of permanent access to water for some of the stallions. The welfare issues recorded in mares were behavior-related and mirrored problems in the human-horse relationship. The source of fear and aggression towards humans could be explored further in an observational study (by monitoring the attitude of the workers during everyday horse handling), following which stud farm personnel could be trained to adopt better practices in this regard.

The fact that the stallions were friendlier towards humans and more indifferent than fearful shows that intensive and correct human handling can greatly improve the human-horse relationship. A good human-horse relationship would result in safer and better horse-handling. It would increase trust on both sides, which in turn would make owners more likely to change their management systems to provide stallions with access to free exercise. 

## Figures and Tables

**Table 1:** The welfare parameters assessed for each of the five freedoms and the scores given [14].

Freedom	Parameter (Score)
Freedom from hunger and thirst	Body condition score (assessed on a 5-point scale and scored 0 for emaciated and also obese conditions which endanger the health/life of the horse; 1 for improper body condition, e.g., thin and fat; 2 for good body condition) Water provision (0—once or two times per day; 1—three or more times per day; 2—unlimited access)
Freedom from discomfort	Fecal soiling on the rump and ventral-lateral abdomen (0—dirty; 1—clean) Hip point lesions (0—presence of skin lesion; 1—scars, thickened skin with missing hair; 2—absence of hip point lesions)
Freedom from pain, injury or disease	Hair coat condition (0—abnormal hair coat on extended body areas; 1—abnormal hair coat on limited body areas; 2—normal hair coat) Hair quality in the mane/tail (0—abnormal; 1—normal) Body lesions (0—severe; 1—superficial; 2—absent) Lower leg lesions (0—severe; 1—superficial; 2—absent) Lip corner lesions (0—presence of at least one lesion; 1—absence of lesion) Lesions at harness contact points (0—presence of at least one disruption of the skin integrity; 1—missing hair at the harness contact points but without wounds; 2—absent) Swollen tendons/joints (0—swollen tendons and joints; 1—swollen tendons or joints; 2—absence of swellings in tendons or joints) Hoof horn quality (0—abnormal; 1—normal) Hoof shape (0—abnormal; 1—normal) Sole surface (0—abnormal; 1—normal) Hoof walls too long or too short (0—too long/short; 1—proper length) Inadequate horseshoes (0—yes; 1—no) Gait (0—lame; 1—abnormal; 2—normal) Dyspnea (0—presence; 1—absence) Cough (0—presence; 1—absence) Nasal discharge (0—presence; 1—absence) Diarrhea (0—presence; 1—absence) Ocular discharge (0—presence of muco-purulent or purulent ocular discharge; 1—presence of serous ocular discharge; 2—absence of ocular discharge) Vision (0—absence of vision in both eyes; 1—absence of vision in one of the eyes; 2—presence of vision in both eyes) Dental check (0—the teeth were never checked; 1—any dentistry intervention at least once during the life of the horse)
Freedom to express normal behavior	Company of its own kind (0—absence of social contact; 1—possibility of social contact with animals from other species; 2—existence of social contact with other horse) Access to unrestricted, free exercise (0—no access to free exercise; 1—limited access, being tethered outside; 2—possibility to free exercise in a space of minimum 20/10 m, minimum 1 hour per day; 3—possibility for free exercise in a space that allows minimum 5 steps at gallop in minimum 2 directions for a minimum of 1 hour per day)
Freedom from fear and distress	General alertness (0—apathetic/depressed; 1—alert) Response to the assessor approaching (0—aggressiveness; 1—fear/avoidance; 2—indifference; 3—friendliness) Response to the assessor walking besides (0—aggressiveness; 1—fear/avoidance; 2—indifference; 3—friendliness) Response to the touch of the assessor (0—aggressiveness; 1—fear/avoidance; 2—indifference; 3—friendliness)

**Table 2:** The classification of the individual welfare scores in qualitative categories of welfare.

Individual Welfare Score	Welfare Categories
0–25	Not Classified
26–35	Acceptable
36–45	Enhanced
46–50	Excellent

**Table 3:** The prevalence of parameters assessed for the investigation of freedom from hunger and thirst in each breeding horse category.

Parameter	Percentage of Stallions (n = 330)	Percentage of Broodmares (n = 365)	*p*-Value
Body condition score (improper)	12.73 (42)	20.00 (73)	0.657
Water provision (limited access)	20.00 (66)	00.00	0.303

If *p* value is less than 0.05 the difference between horse categories is significant.

**Table 4:** The prevalence of health parameters assessed in each breeding horse category.

Parameter	Percentage of Stallions (n = 330)	Percentage of Broodmares (n = 365)	*p-*Value
Abnormal hair coat condition	12.73 (42)	7.67 (28)	0.114
Abnormal hair quality in the mane/tail	10.91 (36)	16.71 (61)	0.507
Body lesions	12.73 (42)	10.41 (38)	0.422
Lip corner lesions	5.76 (19)	0.00	0.082
Lesions at harness contact points	7.27 (24)	3.29 (12)	0.731
Lower leg (foot) lesions	15.45 (51)	7.40 (27)	0.234
Swollen tendons/joints	23.64 (78)	1.64 (6)	**0.002**
Abnormal hoof horn quality	28.48 (94)	1.37 (5)	**0.001**
Abnormal hoof shape	7.57 (25)	6.30 (23)	0.708
Abnormal sole surface	9.39 (31)	0.00	0.072
Hoof walls too long or too short	26.06 (86)	18.90 (69)	0.144
Inadequate horseshoes	0.00	0.00	-
Abnormal gait	64.85 (214)	7.40 (27)	**0.0001**
Dyspnea	16.06 (53)	0.00	**0.008**
Cough	6.36 (21)	0.00	0.104
Nasal discharge	7.57 (25)	3.01 (11)	0.412
Ocular discharge	6.97 (23)	0.00	0.093
Diarrhea	0.00	0.00	-
Abnormal vision	4.85 (16)	2.47 (9)	0.713
Dental check (At least one)	6.36 (21)	6.85 (25)	0.806

For *p*-values less than 0.05 (bold values), difference between horse categories are significant.

**Table 5:** The prevalence of parameters assessed for the investigation of freedom to express normal behaviors in each breeding horse category.

Parameter	Percentage of Stallions (n = 330)	Percentage of Broodmares (n = 365)	*p*-Value
Company of their own kind			
None	0.00	0.00	-
Other mammals	0.00	0.00	-
Other horses	100.00 (330)	100.00 (365)	-
Access to unrestricted, free exercise			
No access to free exercise	100.00 (330)	0.00	0.0001
Limited access	0.00	0.00	-
Small space	0.00	0.00	-
Sufficient space	0.00	100.00 (365)	0.0001

For *p*-values less than 0.05, difference between horse categories are significant.

**Table 6:** The prevalence of behavioral parameters assessed in each breeding horse category.

Parameter	Percentage of Stallions (n = 330)	Percentage of Broodmares (n = 365)	*p*-Value
General alertness			
Apathetic/depressed	5.15 (17)	0.00	0.968
Alert	94.85 (313)	100.00 (365)	0.255
Response to the approach of the assessor			
Aggressiveness	0.00	0.00	-
Fear/avoidance	24.85 (82)	35.07 (128)	0.118
Indifference	32.12 (106)	25.48 (93)	0.267
Friendliness	43.03 (142)	39.45 (144)	0.501
Response to the assessor walking besides the horse			
Aggressiveness	0.00	0.00	-
Fear/avoidance	29.09 (96)	37.53 (137)	0.752
Indifference	21.51(71)	18.63 (68)	0.843
Friendliness	49.4 (163)	43.84 (160)	0.877
Response to the assessor touching the horse			
Aggressiveness	1.51(5)	4.66 (17)	0.227
Fear/avoidance	35.15 (116)	43.01 (157)	0.802
Indifference	16.67 (55)	15.62 (57)	0.947
Friendliness	46.67 (154)	36.71 (134)	0.715

For *p*-values less than 0.05, difference between horse categories are significant.

**Table 7:** The descriptive statistical parameters of the individual welfare scores in each breeding horse category.

Parameter	Breeding Stallions (n = 330)	Broodmares (n = 365)
Mean	39.45	45.00
Standard error of the mean	0.18	0.17
Median	39.00	45.00 *
Minimum	33.00	38.00
Maximum	45.00	50.00

* *p* < 0.05.
